# Inattentional Blindness and Individual Differences in Cognitive Abilities

**DOI:** 10.1371/journal.pone.0134675

**Published:** 2015-08-10

**Authors:** Carina Kreitz, Philip Furley, Daniel Memmert, Daniel J. Simons

**Affiliations:** 1 Institute of Cognitive and Team/Racket Sport Research, German Sport University Cologne, Cologne, Germany; 2 Department of Psychology, University of Illinois at Champaign-Urbana, Champaign, Illinois, United States of America; University of Bath, UNITED KINGDOM

## Abstract

People sometimes fail to notice salient unexpected objects when their attention is otherwise occupied, a phenomenon known as inattentional blindness. To explore individual differences in inattentional blindness, we employed both static and dynamic tasks that either presented the unexpected object away from the focus of attention (spatial) or near the focus of attention (central). We hypothesized that noticing in central tasks might be driven by the availability of cognitive resources like working memory, and that noticing in spatial tasks might be driven by the limits on spatial attention like attention breadth. However, none of the cognitive measures predicted noticing in the dynamic central task or in either the static or dynamic spatial task. Only in the central static task did working memory capacity predict noticing, and that relationship was fairly weak. Furthermore, whether or not participants noticed an unexpected object in a static task was only weakly associated with their odds of noticing an unexpected object in a dynamic task. Taken together, our results are largely consistent with the notion that noticing unexpected objects is driven more by stochastic processes common to all people than by stable individual differences in cognitive abilities.

## Introduction

When their attention is otherwise engaged, people sometimes fail to notice a salient and fully visible, but unexpected object or event, a phenomenon known as *inattentional blindness*. Inattentional blindness can be viewed as a byproduct of attentional selection: Our ability to focus attention enables us to ignore irrelevant or distracting information, but it occasionally leads us to miss items that we might have wanted to experience [1–4].

Many variations in the nature of the unexpected object influence noticing rates: size [5], color [6], semantic content (e.g., [7,8]), and distance from the attentional focus [9,10]. Moreover, the primary-task demands (i.e. current cognitive load; [4,11]) and top-down factors like the observer’s goals or attentional set [12–14] contribute to inattentional blindness.

Although many aspects of the situation affect noticing, it is less clear whether some people are more prone to inattentional blindness than others. Typically, establishing stable individual differences in performance requires repeated tests of the same participants on the same task. Unfortunately, in inattentional blindness tasks, once people know that an object might appear, it will no longer be entirely unexpected, meaning that they might devote some resources to its detection. Consequently, most inattentional blindness studies include only one critical trial for each participant.

Rather than testing the same participants repeatedly, individual-difference studies of inattentional blindness have instead sought correlates of noticing or explored group differences. For example, studies of group differences find greater noticing by people on the autism spectrum [15] and expert athletes [16], and they find reduced noticing by the elderly [17] and those who have consumed alcohol [18]. Studies predicting noticing from cognitive abilities have focused most extensively on working memory capacity, but the results have been mixed and inconclusive. Some studies suggest greater noticing by those with higher working memory capacity [19–22], but other, larger studies find no overall correlation [23,24] (Seegmiller et al. [24] found no overall correlation, but did find an association when restricting analysis to a small subset of participants) or only see an association with a particular primary task [7].

The inconsistencies might simply reflect sampling noise or they could result from differences in the task used to induce inattentional blindness. Inattentional blindness tasks can be clustered into two categories, those governed by the limits of spatial attention and those driven by more central limits on attention [25]. For example, the spatial proximity of an unexpected object to the attended region affects noticing rates [9,10], suggesting a role for spatial selection. But, people also fail to notice unattended objects that are near to, or even overlap with, attended objects [4,9,26], suggesting that spatial limits alone do not drive inattentional blindness.

Perhaps individual differences in cognitive resources will more reliably predict noticing for inattentional blindness tasks that draw on similar resources. For example, working memory or cognitive capacities may contribute to noticing more for those inattentional blindness tasks that are governed by central limits whereas individual differences in spatial attention may predict noticing for spatially-driven tasks. We explored whether controlling for the type of inattentional blindness task might yield more consistent individual differences in noticing. We hypothesized that individual differences in working memory capacity should contribute to noticing when the task is driven by central factors (e.g. expectations or cognitive load), whereas individual differences in attention breadth should contribute more to noticing when inattentional blindness is induced by diverting attention spatially.

In two studies with large, independent samples we assessed inattentional blindness using a static task (Study 1 and 2) and a dynamic task (Study 2). For each type of task, we manipulated the spatial position of the unexpected object. In the Near condition, the unexpected object appeared within or near the area that participants needed to focus attention to complete the primary task. For this condition, inattentional blindness should be determined more by cognitive limits than spatial ones. In the Far condition, the unexpected stimulus appeared away from the attended area, meaning that inattentional blindness could result, at least in part, from spatial limits. We explored individual differences in centrally- and spatially-induced inattentional blindness by predicting noticing using multiple measures of cognitive and attention abilities.

## Study 1

Study 1 investigated the hypothesis that individual differences in working memory capacity would predict centrally-induced inattentional blindness whereas differences in attention breadth would predict spatially-induced inattentional blindness. Specifically, we predicted a link between working memory capacity and noticing for objects near the focus of attention, because failures to notice in such cases might result from central capacity limits. And, we predicted a link between attention breadth and noticing for unexpected objects appearing away from the focus of attention, because inattentional blindness in such cases might result from spatial limits.

Once participants have learned of the existence of an unexpected object, subsequent trials measure their ability to detect now-expected objects when performing the primary task. We predicted that individual differences in working memory capacity would correlate with noticing on these “divided-attention” trials because people with greater working memory capacity should be able to allocate their attentional resources more flexibly [27,28].

It has been argued that different processes might underlie the capability to inhibit known distraction and the tendency to stay unaware of *unexpected* stimulation [29] (see also [30]). Although individual differences in the ability to ignore known distractions have been studied extensively [31], to our knowledge, few studies have examined whether differences in that ability are related to proneness to inattentional blindness. We aimed at shedding further light on that issue by assessing whether the ability to actively inhibit distractors in a Flanker task is related to the tendency to miss unexpected objects in an inattentional blindness task. Intuitively, we might expect that people who can better filter known distractions from awareness would also be less likely to notice unexpected objects. However, superior performance on focused attention tasks does not correlate with noticing [32] and noticing does not appear to be related to performance on inhibitory control tasks like the Stroop task [20]. Consequently, we predicted no relationship between inattentional blindness and the ability to ignore known distractors in an Eriksen Flanker task.

Methods, hypotheses, data preparation, and analyses for Study 2 were pre-registered before the data collection started. Although Study 1 was not formally pre-registered, we had specified our hypotheses in a prior grant proposal, and our procedures and predictions followed the same plan that we later pre-registered for Study 2. Data from the working memory and attention breadth tasks in Study 1 were presented in a previous paper that examined the relationship between these two constructs [33]. For the purpose of completeness and transparency, we identified and described all of the collected measures both in this paper and in the earlier paper. But, the questions addressed and the analyses reported in each paper are distinct; the earlier paper did not present data from the Flanker task or the inattentional blindness task, and it did not explore links between inattention blindness and any of the measures. We refer readers to the earlier paper for a discussion of the links between attention breadth and working memory.

### Method

#### Ethics statement

The reported studies were reviewed and approved by the ethics committee of the German Sport University Cologne. All participants gave written informed consent prior to their inclusion in the study and they were debriefed afterwards.

#### Participants

A total of 123 participants gave written informed consent, reported normal or corrected-to-normal vision, and received 13 € for their participation. Data from two of these participants were excluded from the analysis because they reported that they had anticipated the unexpected object. Four other participants either failed to report the unexpected object or could not identify either its position or its shape when looking for the critical object was their only task (i.e., with full attention). In such cases, the failure to notice an unexpected object becomes ambiguous because participants might not have followed instructions or they might have other (perceptual) problems that limit their ability to see the object even when they are trying to. In keeping with tradition in the inattentional blindness literature (e.g., [5]) and our analysis plan, data from these participants were excluded from the analysis. Finally, one additional participant was excluded because the thresholding procedure for the line-judgment task could not be completed before the unexpected object appeared. The analyzed data set consisted of the remaining 116 participants (*M* = 22.9 years, *SD* = 4.1 years, 55 female). Of these, 60 participants were in the Near condition and 56 were in the Far condition.

There were no strong associations between inattentional blindness and any of the demographic variables we included. Details regarding these exploratory analyses and the exact statistical values can be found on https://osf.io/mvwih/.

#### Tasks

In addition to an inattentional blindness task, each participant completed a battery of cognitive tasks designed to measure different attention abilities. The battery included three working memory tasks, two attention breadth tasks, a Flanker task, and the German version of a questionnaire measuring cognitive failures in daily life (the CFQ; [34]; original version: [35]). Each is described in detail below, and code for these tasks is available at https://osf.io/tmlgj/. The tasks are also described in our earlier paper focusing on the relationship between working memory and attention breadth [33].

Participants completed a static inattentional blindness task (IB Cross task; [10]) in which they repeatedly judged which of two arms of a briefly presented cross was longer, and following a critical trial, were asked whether they had noticed the appearance of an unexpected shape. In this task, the proximity of the unexpected object to the cross affects noticing rates [10]. We employed two different conditions: On the critical trial, the unexpected object appeared either near the center of the cross (Near condition) or farther away from it (Far condition). Each trial began with a 1000 ms fixation screen, followed by a cross for 200 ms and then by a black-and-white pattern mask for 500 ms. Participants judged which line of the cross, the horizontal one or the vertical one, was longer and responded using a keyboard. Which line was longer was determined randomly on each trial. The longer line always had a length of 6° and the length of the shorter line was adjusted using an adaptive staircase procedure (3–1 method; [36]) for each individual in order to equate the difficulty of the cross task at 79% accuracy. By keeping the size of the longer line constant, the overall extent of the cross was invariant across trials and participants, allowing us to use identical placements for the unexpected object in the Far condition across participants.

Each participant completed ten easy practice trials (smaller arm 4.5°) before the thresholding began. Once the threshold had been determined for an individual, the critical trial occurred immediately and without forewarning. The cross on this critical trial used the threshold value determined for that participant, and a grey square (0.9° x 0.9°; RGB: 128,128,128) appeared along with the cross for the entire 200 ms. The square was always presented on one of the imaginary 45° lines bisecting the quadrants defined by the cross, with the particular quadrant chosen randomly for each participant. In the Near condition, the square appeared 2° from the center of the cross. In the Far condition, it appeared 7° from the center of the cross. After reporting which line they thought was longer, participants were asked if they had seen anything other than the cross that had not been present before. They were then asked how confident they were of their answer (very, somewhat, not at all), where the additional object had appeared (upper right, lower right, lower left, upper left), and which shape it had been (six choices). They were asked to guess if they had not noticed anything. Participants were coded as inattentionally blind if they did not report noticing the unexpected object or claimed to have seen something but could not define either its location or its shape.

After these questions, participants were told that the experiment would continue with more trials of the line-judgment task. Following three “normal” line-judgment trials (at threshold length), the grey square appeared for a second time and at the same distance from fixation as on the critical trial, although the quadrant was again chosen randomly. After reporting which arm was longer, participants answered the same questions that had followed the critical trial. Following the divided-attention trial, participants completed a final trial on which they were told to not perform the cross task (full-attention trial). The location (quadrant) of the additional square was again chosen randomly, but it was positioned at the same distance from the center of the cross as on previous trials. Participants were not asked to perform the line judgment, but were given the same questions about the additional square.

The three working memory measures included the automated version of the operation span task (Aospan; [37]) and both a verbal and a spatial 2-back task [38,39]. These tasks measure both memory storage and attention-control mechanisms [28,40], and together they provide converging but distinct [41] estimates of individual differences in working memory. In the Aospan task, participants solved simple math problems while remembering sets of letters. The task included a total of 15 trials consisting of 3 trials with 3, 4, 5, 6, and 7 letters. The resulting Aospan score is the total number of letters recalled across all trials for which the participant correctly remembered the whole letter sequence. In the verbal 2-back task (2-Back-Identity), participants viewed a sequence of 100 letters (1.7° of visual angle, drawn from the set: C, F, K, M, P, S, W, X) presented at fixation, and pressed a key whenever the current letter matched the one presented two items earlier in the stream. Across the 100 letters in the sequence, there were 25 2-back matches. In exactly five cases, the current item matched the one presented immediately before it, and in five cases, the current item matched that presented three items earlier (distractor items). Prior to this critical sequence, participants completed a 20-letter practice sequence. In the 2-Back-Spatial task, the letters were replaced with circles (2° diameter) that could appear at one of 8 possible spatial locations (equally distributed on an imaginary circle centered on fixation with a diameter of 15°). The participant’s task was to respond whenever the position of the current circle matched that from two earlier in the sequence. For both 2-back tasks, we used Pr (hits—false alarms; see [42]) as our measure of working memory capacity.

The two attention breadth measures included a useful-field-of-view task (UFOV; adapted from [43]) and a breadth-of-attention task (BoA; adapted from [44,45]). Each measures the maximum extent to which participants can spread their attention when attending simultaneously to tasks at two distinct locations. Both tasks measure the spatial distribution of attention and not differences in peripheral visual acuity [43,44], and both have been used to study individual differences [44,46].

**Fig 1:**
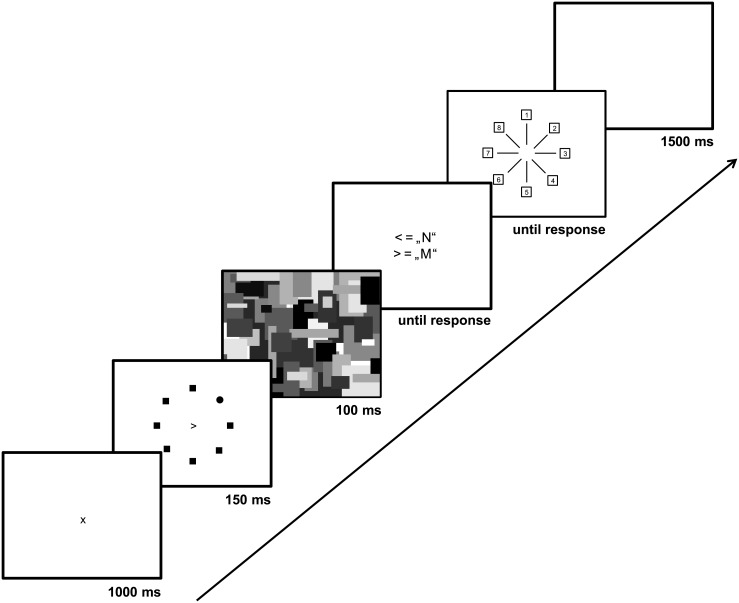
The useful-field-of-view task (UFOV). Sequence of events in a trial from the attention breadth measure useful-field-of-view (UFOV). One possible stimulus configuration was randomly picked for this display.

In our version of the UFOV task, participants tried to detect a peripherally presented circle among square distractors while also judging whether a central figure (< or >) pointed left or right (see Fig 1). The circle appeared at one of 8 locations on an imaginary circle that was centered at fixation and had a radius of 6.3°, 9.5°, or 12.7°. On each trial, a fixation cross appeared for 1000 ms followed by the stimuli for 150 ms and then by a 100 ms pattern mask. Participants pressed keys to indicate the directionality of the central figure and the location of the peripheral target. Participants completed 68 practice trials and 120 experimental trials (40 at each radius, with 5 at each position on the circle). Each trial was followed by a 1500 ms blank interval and trial order was randomized for each participant.

**Fig 2:**
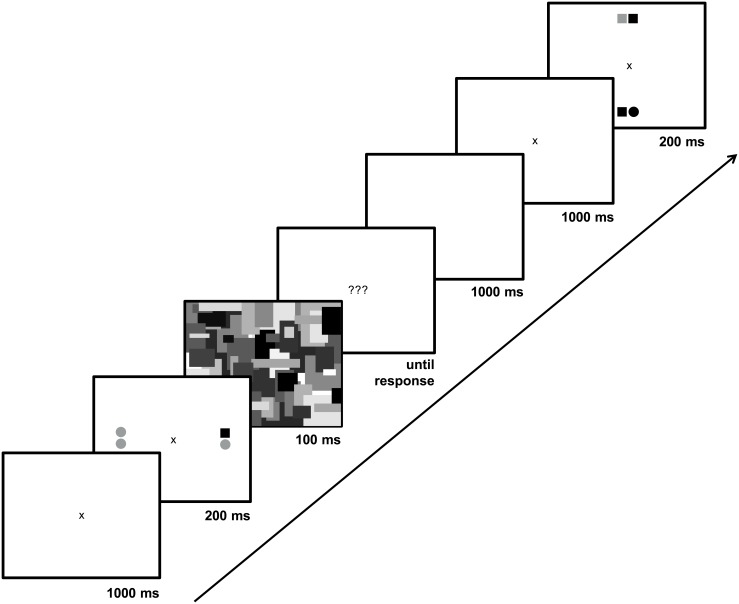
The breadth-of-attention task (BoA). Sequence of events from the breadth-of-attention task (BoA). Two possible stimulus configurations were randomly picked for this display.

In the BoA task, participants fixated a central cross for 1000 ms and then judged the total number of gray circles appearing among two spatially separated pairs of shapes that appeared for 200 ms (circles and squares that were gray or black; see Fig 2). The shapes were followed by a 100 ms pattern mask. We used adaptive thresholding (3–1 procedure; [36]) separately for horizontally and vertically separated clusters (with trials randomly interleaved), adjusting the separation between the clusters until we reached a threshold of 79% accuracy for each participant on each dimension. We used the average of the horizontal and vertical threshold values as a measure of that individual’s attention breadth.

The Eriksen Flanker task measures the ability to inhibit distractor stimuli [47,48]. In our implementation of the Flanker task, participants first viewed a fixation cross for 500 ms, followed by an array (5.7° wide) of five Hs and Ss for 500ms. Participants judged as quickly as possible whether the central letter was an H or S, while the surrounding letters were congruent (SSSSS or HHHHH) on half of the trials and incongruent (SSHSS or HHSHH) on the other half. All trial types were randomly intermixed, and after 20 practice trials, participants completed 100 experimental trials. The primary measure of Flanker-task performance was the percentage increase in response time on incongruent trials relative to congruent ones, for correct responses only: [(incongruent-congruent)/congruent]*100.

Participants completed the German version of the Cognitive Failures Questionnaire (CFQ) to assess whether cognitive failures in daily life are associated with failures of awareness of unexpected objects. High scores in this questionnaire indicate a higher tendency to cognitive failures in daily life.

#### Procedure

A chin rest (NovaVision, Magdeburg, Germany) was used for the inattentional blindness task, the BoA, and the UFOV. It was positioned 50 cm from a 24-inch display (resolution: 1920 x 1080 pixels, controlled by an Esprimo 710 3.3 GHz Core i3-3220 computer). For all other tasks, participants sat approximately 50 cm from the display. The Aospan was presented using E-Prime 2.0 (Psychology Software Tools, Pittsburgh, PA) and all other tasks were presented using Presentation (Neurobehavioral Systems, Berkeley, CA). Participants used a keyboard or, in case of the Aospan, a mouse to respond.

Participants were tested alone or in pairs. When participants were tested in pairs, they were separated by dividers that prevented them from seeing each other. Instructions were delivered on the screen prior to each task, but participants were also encouraged to ask questions before starting. The inattentional blindness task was always completed first, followed by the other cognitive tasks (three working memory tasks, two attention breadth tasks, Flanker task) in a randomized order for each participant. Whenever two participants completed the study in the same session, they were given the same randomized task order to minimize interruptions and distraction, and the experimenter waited for both participants to complete each task before starting the next task. A general questionnaire (retrieving demographics, anticipation of the unexpected object, and familiarity with inattentional blindness) was administered after the inattentional blindness task and the CFQ was administered after the completion of three cognitive tasks. The entire testing session took approximately two hours.

### Results & Discussion

Due to technical errors, data were missing for some participants in some tasks (six subjects in the 2-Back-Identity task, four subjects in the 2-Back-Spatial task, three subjects in the BoA task, and three subjects in the Flanker task). One participant did not fully complete the CFQ. The correlational analyses included all participants for whom we had data for both measures. All of the reported significance levels are based on two-tailed tests.

As noted earlier, we analyzed the relationships between and among the working memory and attention breadth measures in a separate publication (see [33]). That paper reported an association between each of the working memory measures and the two attention breadth measures, with the two constructs sharing a substantial amount of variance. In it, we discussed the implications of those findings for our understanding of working memory. In contrast, the present paper investigates the cognitive mechanisms underlying failures of awareness and we focus on the relationship between the cognitive measures and inattentional blindness.

Given that we did not have separate hypotheses for the relationship between inattentional blindness and the individual working memory or attention breadth measures, we formed composite measures of both cognitive constructs to use in predicting noticing rates. This seemed reasonable as the working memory measures were moderately to highly intercorrelated with each other (2-Back-Identity and 2-Back-Spatial: *r* = .52 [.37, .64], 2-Back-Identity and Aospan: *r* = .30 [.12, .46], 2-Back-Spatial and Aospan: *r* = .21 [.03, .38]) and the two attention breadth measures were highly correlated (*r* = .50 [.35, .63]). Nevertheless, as there has been research suggesting that n-back tasks and complex-span tasks do not reflect a single construct [41,49], we additionally report correlations of each single measure (Table 1).

**Table 1:** Correlations (point biserial) among noticing of an unexpected object (all participants, Near condition, Far condition) and the cognitive tests; Study 1.

	ALL		NEAR		FAR	
	Notice	Notice	Notice	Notice	Notice	Notice
	(critical)	(divAtt)	(critical)	(divAtt)	(critical)	(divAtt)
Working Memory	.08	-.01	.24	.07	-.08	-.06
	[-.10, .26]	[-.19, .17]	[-.02, .47]	[-.19, .32]	[-.34, .19]	[-.32, .21]
	N = 116	N = 116	N = 60	N = 60	N = 56	N = 56
2-Back-Identity	.05	-.10	.23	-.03	-.13	-.12
	[-.14, .24]	[-.28, .09]	[-.04, .47]	[-.29, .24]	[-.38, .14]	[-.38, .15]
	N = 110	N = 110	N = 56	N = 56	N = 54	N = 54
2-Back-Spatial	.04	.09	.04	.18	.06	.03
	[-.15, .22]	[-.10, .27]	[-.22, .30]	[-.09, .42]	[-.21, .32]	[-.24, .29]
	N = 112	N = 112	N = 57	N = 57	N = 55	N = 55
Aospan	.07	-.06	**.26**	-.03	-.15	-.08
	[-.11, .25]	[-.24, .12]	**[.01, .48]**	[-.28, .23]	[-.40, .12]	[-.34, .19]
	N = 116	N = 116	N = 60	N = 60	N = 56	N = 56
Attention Breadth	.14	-.01	**.32**	.06	.00	.01
	[-.04, .31]	[-.19, .17]	**[.07, .53]**	[-.20, .31]	[-.26, .26]	[-.25, .27]
	N = 116	N = 116	N = 60	N = 60	N = 56	N = 56
BoA	.10	-.04	**.29**	.02	-.10	-.02
	[-.09, .28]	[-.22, .15]	**[.04, .51]**	[-.24, .27]	[-.36, .18]	[-.29, .25]
	N = 113	N = 113	N = 60	N = 60	N = 53	N = 53
UFOV	.14	.03	**.26**	.09	.05	.03
	[-.04, .31]	[-.15, .21]	**[.01, .48]**	[-.17, .34]	[-.22, .31]	[-.24, .29]
	N = 116	N = 116	N = 60	N = 60	N = 56	N = 56
Flanker	.08	-.09	.02	-.19	.13	-.04
	[-.11, .26]	[-.27, .10]	[-.24, .28]	[-.43, .07]	[-.14, .38]	[-.30, .23]
	N = 113	N = 113	N = 59	N = 59	N = 54	N = 54
CFQ	**-.20**	**.20**	-.22	.15	-.23	.20
	**[-.37, -.02]**	**[.02, .37]**	[-.45, .04]	[-.11, .39]	[-.47, .04]	[-.07, .44]
	N = 115	N = 115	N = 60	N = 60	N = 55	N = 55

*Note*. In addition to the single cognitive measures the composite working memory measure and the composite attention breadth measure are depicted. The lower and upper bounds of the 95% confidence interval are shown in square brackets. *N* = number of cases contributing to each correlation. Correlations that are significant (α = 0.05; two-tailed) appear in bold, although the alpha level has not been corrected for multiple tests.

To create the composite measures we first standardized the scores for each task across individuals and then averaged each participant’s z-scores across measures for each construct to provide a single working memory z-score and a single attention breadth z-score for each participant. If a participant was missing a score on one of the measures, we formed the composite measure from the remaining measures.

Table 2 provides descriptive statistics and test-retest reliabilities (19 participants were retested after approximately 2 weeks) for all of the cognitive-performance measures. All tasks showed substantial variability across individuals, and noticing rates in the inattentional blindness task were not near ceiling or floor levels, making an exploration of individual differences feasible. Replicating prior evidence that spatial proximity to the focus of attention affects noticing rates in an inattentional blindness task [10], the unexpected object was noticed more often in the Near condition than in the Far condition, both on the critical trial (Near: 46.7%, Far: 28.6%, *χ* ^*2*^ *(1)* = 4.03, *p* = .045, risk ratio = 1.63) and on the divided-attention trial (Near: 76.7%, Far: 57.1%, *χ* ^*2*^ *(1)* = 5.01, *p* = .025, risk ratio = 1.34).

**Table 2:** Descriptive data.

	Study 1			Study 2			
	*N*	Mean	*SD*	*N*	Mean	*SD*	*r*
2-Back-Identity	110	17.39	4.78	197	18.28	4.59	0.78
2-Back-Spatial	112	16.04	5.52	197	17.05	5.19	0.49
Aospan	116	37.06	17.85	198	40.18	16.27	0.69
BoA	113	286.92	59.86	198	284.69	57.10	0.77
UFOV	116	0.77	0.18	198	0.77	0.15	0.87
Flanker	113	9.81	4.39	197	10.22	5.04	0.52
CFQ	115	42.37	11.42	198	45.55	11.98	0.80
Navon	-	-	-	198	9.96	13.09	0.59
Navon-Switchspeed	-	-	-	198	21.87	12.21	0.33

*Note*. *N* = number of cases in the analysis, *SD* = standard deviation, *r* = test-retest reliability, mean of 2-Back-Identity and 2-Back-Spatial in Pr, mean of Aospan refers to the Ospan score, mean of BoA refers to the averaged threshold (in pixels), mean of UFOV represents the proportion of correct responses, mean of the Flanker task depicts the percent increase in response times to incongruent trials, mean of the CFQ represents the item scores added up, mean of the Navon task depicts the percent increase in response times to local stimuli, and mean of the Navon-Switchspeed task depicts the percent increase in response time to incongruent stimuli. Data from the three working memory tests and the two attention breadth tests of Study 1 were presented previously, in a separate paper that examined relationships among those measures (see Kreitz et al., 2014).

Table 1 shows the correlations between each of the cognitive measures and noticing in the inattentional blindness task. Collapsing across the Near and Far conditions, none of the cognitive measures was significantly associated with noticing of the unexpected object in either the critical trial or the divided-attention trial. The CFQ was negatively correlated with noticing on the critical trial and positively correlated with noticing on the divided-attention trial. A negative correlation suggests that people who are more prone to cognitive failures in daily life might also be more likely to miss an unexpected object. However, the reversal of this pattern on the divided-attention trial is harder to interpret and signals the need for caution in drawing strong conclusions about this association.

**Fig 3:**
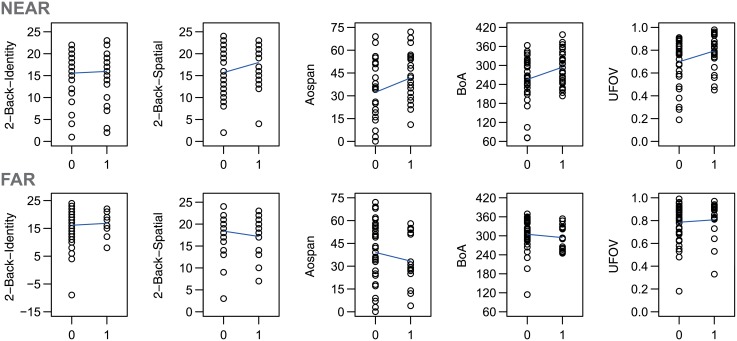
Scatterplots for Study 1. Scatter plots of the relationships between inattentional blindness (0 = miss, 1 = notice) and the working memory and attention breadth measures in Study 1. The plots were prepared separately for the Near and the Far condition. The y-axes depict the test scores for each measure as described in the method section. Each circle represents a single participant. The blue lines depict the linear regression lines for each relationship.

We hypothesized that working memory measures might predict noticing in the Near condition but not the Far condition, and that attention breadth measures would predict noticing in the Far condition but not the Near condition. Inconsistent with our hypothesis, none of the measures was associated with noticing in the Far condition. Consistent with our predictions, Aospan was correlated with noticing in the Near condition. However, the other working memory measures were not, and the composite working memory measure was not significant either. Moreover, both attention breadth measures and the composite attention breadth measure were also associated with noticing in the Near condition. Note, though, that none of these correlations would have been statistically significant following correction for multiple tests, so they should be treated as suggestive and they require replication with a larger sample. Scatter plots of the relationships between inattentional blindness and the working memory and attention breadth measures are depicted separately for the Near and the Far condition in Fig 3.

To determine whether the cognitive measures in combination could effectively predict noticing of the unexpected object on the critical trial, we conducted two logistic regression analyses, one for the Near condition and one for the Far condition (see Table 3). In each case, we predicted noticing from the working memory and attention breadth composite measures, the Flanker task, and the CFQ. The working memory and attention breadth measures were included first (using the enter method), and the CFQ and Flanker measures were entered next. This approach allowed us to evaluate the hypothesized role of working memory and attention breadth in a separate first block.

**Table 3:** Results of the binary logistic regression with simultaneous entry in two blocks (SE in parentheses); Study 1.

NEAR						
	Variables	*B* (*SE*)	Wald	Exp(*B*)	Exp(*B*) lower	Exp(*B*) upper
	Constant	-0.07 (0.28)	0.07	0.93		
	Working Memory	0.42 (0.41)	1.06	1.53	0.68	3.43
	Attention Breadth	0.66 (0.38)	2.99	1.94	0.92	4.09
	*R* ^*2*^ = .*12 (Cox & Snell)*, *Model*: *χ* ^*2*^ *(2) = 7*.*47*, *p <* .*05*					
	Constant	2.13 (1.56)	1.86	8.41		
	Working Memory	0.45 (0.42)	1.16	1.57	0.69	3.57
	Attention Breadth	0.68 (0.40)	2.91	1.97	0.90	4.31
	CFQ	-0.05 (0.03)	2.82	0.95	0.90	1.01
	Flanker	-0.01 (0.07)	0.02	0.99	0.86	1.14
	*R* ^*2*^ = .*16 (Cox & Snell)*, *Model*: *χ* ^*2*^ *(4) = 10*.*48*, *p <* .*05*					
FAR						
	Variables	*B* (*SE*)	Wald	Exp(*B*)	Exp(*B*) lower	Exp(*B*) upper
	Constant*	-0.93 (0.32)	8.74	0.39		
	Working Memory	-0.24 (0.46)	0.27	0.79	0.32	1.93
	Attention Breadth	0.04 (0.46)	0.01	1.04	0.42	2.55
	*R* ^*2*^ = .*01 (Cox & Snell)*, *Model*: *χ* ^*2*^ *(2) = 0*.*31*, *p =* .*86*					
	Constant	0.26 (1.31)	0.04	1.29		
	Working Memory	-0.37 (0.48)	0.62	0.69	0.27	1.75
	Attention Breadth	0.01 (0.47)	0.00	1.01	0.40	2.53
	CFQ	-0.05 (0.03)	3.15	0.95	0.90	1.01
	Flanker	0.08 (0.07)	1.17	1.08	0.94	1.25
	*R* ^*2*^ = .*09 (Cox & Snell)*, *Model*: *χ* ^*2*^ *(4) = 5*.*05*, *p =* .*28*					

*Note*. Both regressional analyses (Near, Far) were performed in two separate blocks. The variables of the first block are depicted first and the variables of the whole model, including the second block, are depicted below it. The upper and the lower bounds of the 95% confidence interval of Exp(*B*) are depicted as well.

*p < .05

Consistent with the correlational analysis, for the Far condition, the working memory and attention breadth composites explained almost none of the variance in noticing (approximately 1%), and including the CFQ and Flanker measures as predictors did not improve the model, either. Again, this finding does not support our hypothesis that attention breadth would predict noticing in the Far condition. One possible explanation for the lack of a relationship might be that the maximum spatial separation at which people can perform two tasks, as it is measured by the UFOV and the BoA, is not related to the direction and spread of attention within the IB task. The cross task does not require a wide spread of attention and people might not distribute their attention more broadly here even if they could.

For the Near condition, the composite working memory and attention breadth measures jointly accounted for about 12% of the variance. However, neither measure improved the model significantly on its own, and adding the CFQ and Flanker task did not significantly improve the model. This result mirrors that of the correlational analysis, suggesting only a relatively weak relationship between centrally-induced inattentional blindness and individual differences in working memory and attention breadth.

The lack of a link between the Flanker task and noticing in any of these analyses suggests that the ability to actively inhibit a known distractor is not related to missing unexpected items when focusing attention. This finding is consistent with the lack of a relationship between inattentional blindness and performance on the Stroop task [20] as well as with the lack of a relationship between the ability to perform the primary task and noticing in a dynamic tracking task [32]. Together, these findings suggest that inhibitory processing is not central to the failure to detect unexpected objects in an inattentional blindness task.

We had also hypothesized that working memory capacity would predict performance on the divided-attention trial because working memory capacity typically has been linked to greater attentional control and to more flexible and goal-oriented allocation of attention [27,28]. In the divided-attention trial, participants perform the primary task while presumably also devoting some resources to the detection of the extra shape. Contrary to our prediction, neither the composite working memory measure nor the separate measures were significantly correlated with noticing on the divided-attention trial in either the Near or Far condition (Table 2).

## Study 2

Study 2 was designed to replicate and extend the findings of Study 1 with a larger sample size, a second inattentional blindness task, and additional cognitive measures. Specifically, we added a dynamic inattentional blindness task to explore the possibility that working memory and attention breadth might better predict performance when participants must sustain focused attention continuously for a longer time. In both tasks, we included a Near and Far variant, allowing us to replicate the conditions of Study 1 and to compare performance between comparable conditions in a static and dynamic inattentional blindness task.

By including a second task, we also could explore whether inattentional blindness in one task predicts inattentional blindness in another task. If performance in these two inattentional blindness tasks is uncorrelated, it is less likely that the tendency to notice or miss an unexpected object is a stable aspect of an individual. Instead, the lack of a correlation would imply that noticing on any given inattentional blindness task is a stochastic process, with some probability that any individual will happen to notice. If true, individual differences in other cognitive abilities also probably would not predict inattentional blindness across tasks and situations. To our knowledge, no previous study has examined whether noticing or missing unexpected objects is a stable individual-difference trait. Typically, once a participant knows that an unexpected object might appear, any subsequent trial of the same task no longer tests inattentional blindness. Previous studies have shown that people can miss unexpected objects even if they are familiar with the construct [50] and that they can miss additional objects when they expect a different unexpected object [51], but none have examined whether a person who misses one truly unexpected object is more likely to miss another. We embedded two distinct inattentional blindness tasks in a larger battery of tasks. The tasks differed substantially both in their appearance and in the nature of the primary task, with the dynamic task at the beginning and the static cross task at the end. These procedures helped ensure that participants did not anticipate an unexpected object in the second task.

In addition to the second inattentional blindness task, Study 2 included two variants of the Navon task [52,53], one designed to measure individual differences in global/local attention style and the other to measure the ability to switch quickly between a global and local focus (Navon-Switchspeed). Individual differences in attention style have been linked to the attention blink [54,55], another failure of awareness. We hypothesized that a global style might increase noticing rates in the Far condition whereas a local style might increase noticing rates in the Near condition. We also hypothesized that participants who could switch focus more efficiently might be more likely to notice unexpected objects in the dynamic task given that it requires participants to follow multiple moving objects across a larger display. But, we predicted that switching speed would not predict performance in the static task given that it does not require participants to shift between a local and global focus.

As in Study 1, we explored whether performance on the Flanker task would predict noticing. Although we found no evidence that individual differences in inhibition influenced noticing in the static inattention blindness task in Study 1, the dynamic task used in Study 2 requires participants to ignore some items. In that context, inhibitory control might enhance the ability to focus just on the attended shapes, thereby increasing inattentional blindness.

Study 2 was pre-registered. All hypotheses, procedures, data preparation, and analyses were specified in advance, and data and materials are available at the Open Science Framework (https://osf.io/btlzj/, https://osf.io/mvwih/). Unlike Study 1, none of the data from this study were presented elsewhere.

### Method

#### Participants

A total of 200 participants gave written informed consent, reported normal or corrected-to-normal vision, and received 13 € and a chocolate bar for their participation. Data from two participants were excluded from the analysis because they could not understand the language in the instructions sufficiently well and the session had to be aborted. All participants successfully reported the unexpected object on the full-attention trial of at least one of the inattentional blindness tasks and no participant reported having expected the additional object on *both* of the IB tasks (i.e., for all participants, the additional object was unexpected on at least one of the IB tasks), so the final analyses are based on 198 participants (96 female; mean age = 21.3 years, *SD* = 3.1 years). Data from some participants were excluded for particular tasks, and the reasons and resulting sample sizes are noted in the results.

There were no strong associations between inattentional blindness and any of the demographic variables we included. Details of these and other exploratory analyses can be found at https://osf.io/mvwih/.

#### Materials and procedure

Except as noted, all materials and procedures were identical to those of Study 1. Study 2 added a dynamic inattentional blindness task (IB Motion task) and two variants of the Navon task (described below). The chin rest was used for both inattentional blindness tasks, the BoA, and the UVOF. The IB Motion task was always completed first and the static inattentional blindness task (IB Cross task) was presented last, with the remaining cognitive tasks interspersed between them. The two Navon tasks were always presented in succession, with the standard task first (from the participant’s perspective, they were essentially the same task). Otherwise, the order of the cognitive tasks was randomized for each participant. As in Study 1, whenever two participants took part in the same session, they completed the tasks in the same order. Each participant was assigned to the same condition for both inattentional blindness tasks (Near or Far). The general questionnaire was administered immediately after the IB Motion task and the CFQ was administered after the third cognitive task.

The IB Motion task was adapted from Most et al. [9]. On each trial, observers tracked 4 red Ls and Ts (1.15° x 1.15°) as they moved around a white display window (19.6° x 14.8°), counting the total number of times that any of them touched a black horizontal line. They ignored 4 blue Ls and Ts that also moved about the window. All objects moved linearly and rebounded in a random direction whenever they touched the edge of the window. Whenever a red or blue letter occupied the same space, the red one occluded the blue one. Participants were instructed to fixate a central square throughout each trial. Each trial started with the objects unmoving for 600 ms, followed by 8 s of motion, and then by 300 ms with unmoving objects at the end. Subjects then were prompted to type in their total count.

Each participant first completed three practice trials in which the objects moved at 3.8° per second followed by three more in which they moved at 8.6° per second. After these practice trials, they completed a set of trials in which the speed of the objects was adaptively adjusted (3–1 method; [36]), getting faster when they counted correctly and slower when they counted incorrectly, until reaching the speed at which they could respond accurately 79% of the time. Counts were treated as correct if they were within 10% of the correct answer (rounded up; see [32]). Once the threshold was determined, the critical trial occurred next with no forewarning. On the critical trial, the letters moved at threshold speed and in addition to the Ls and Ts, a light gray cross (0.9° x 0.9°) entered on the right side of the display after 2.9s, traveled horizontally across the display, and exited on the left 2.2 s later. Whether the cross appeared above or below the attended line was determined randomly for each participant. In the Near condition, it was 1.5° from the line, and in the Far condition, it was 5° from the line. After reporting their total count, participants were asked if they had seen anything other than the letters on the preceding trial. Next, they were asked whether the additional object had appeared above or below the line, what color it had been (5-alternative forced choice), and what shape it had been (6-alternative forced choice). If they had not reported seeing anything other than the letters, they were asked to guess in answering these questions. Next, they completed three more trials with objects moving at the threshold speed before the “unexpected” object appeared again (the divided-attention trial). After answering the same set of questions, they experienced one more trial in which they were told not to count the touches (the full-attention trial). For the divided- and full-attention trials, the distance from the line was the same as it had been in the critical trial, but whether it appeared above or below the line was randomized.

In the Navon task, participants viewed centrally presented letters (5.15°) that were composed of a set of identical smaller letters (0.69°), with each horizontal and vertical segment of the larger letter consisting of five smaller letters. Participants were asked to judge whether there was an H or L in the display and to press the corresponding key on the keyboard. On each trial, either the large letter was an H or L or it was composed of all Hs or all Ls. The other stimulus on each trial was an F or T. The stimuli followed a 1500 ms fixation cross and remained visible until the participant had responded. Participants completed 16 practice trials (each stimulus combination twice) followed by 64 experimental trials (each combination 8 times), with trial types randomly intermixed. The primary measure of global/local attentional style was the percent increase in response time to local stimuli over the response time to global stimuli for correct trials: [(local-global)/global]*100.

The stimuli and procedure for the Navon-Switchspeed task were identical to those for the Navon task. The inter-stimulus interval was reduced to 16 ms, and participants completed 161 trials. For 80 of these, the target location was congruent with the preceding trial (a global target followed a global target or a local target followed a local target) and for 80 it was incongruent (a global target followed a local target or a local target followed a global target). The first trial was neither congruent nor incongruent and was not considered in the analysis. Congruent and incongruent trials were randomly intermixed, and the stimulus on each trial was randomly chosen from the set of letter combinations that fit the condition (i.e., from the four global or the four local stimuli). Only correct trials were analyzed and the primary measure (switch speed) was the percent increase in response time for incongruent trials relative to congruent trials: [(incongruent-congruent)/congruent]*100.

### Results & Discussion

For the IB Motion task, three subjects reported having expected an additional object and six either failed to notice the object in the full-attention trial or failed to identify at least two of its features. After excluding data from these nine participants, the analyses included 96 participants in the Near condition and 93 participants in the Far condition for that task. Participants were coded as inattentionally blind if they did not report the unexpected object or claimed to have seen something but could not define at least two of the three features. For the IB Cross task, 13 participants reported expecting an additional object and three either failed to report the object in the full-attention trial or could not identify at least its position or its shape. After excluding data from these 16 participants, analyses of the IB Cross task included 95 participants in the Near condition and 87 participants in the Far condition. Participants were coded as inattentionally blind if they did not report the unexpected object or claimed to have seen something but could not define at least either its location or its shape.

Due to technical errors, data were missing for some participants in some of the cognitive tasks (one subject in the 2-Back-Identity task, one subject in the 2-Back-Spatial task, and one subject in the Flanker task). For correlational analyses, we included all participants who had data for both measures. As in Study 1, we formed a composite working memory measure from the three working memory tasks and a composite attention breadth measure from the two attention breadth tasks (see Table 1 for descriptive statistics and test-retest reliabilities for all measures). All statistical analyses conducted were two-tailed and followed our pre-registered plan.

For the critical trial in the IB Motion task, participants in the Near condition noticed the unexpected object more often (65.6%) than those in the Far condition (36.6%), *χ* ^*2*^ *(1)* = 15.97, *p* < .001, risk ratio = 1.79. Replicating the pattern of Study 1 and of earlier research (Newby & Rock, 1988), participants were also more likely to notice the unexpected object in the Near condition (62.1%) than in the Far condition (34.5%) of the IB Cross task, *χ* ^*2*^ *(1)* = 13.87, *p* < .001, risk ratio = 1.80. The same pattern held for the divided-attention trial of the IB Motion task (Near: 82.3%, Far: 61.3%, *χ* ^*2*^ *(1)* = 10.33, *p* = .001, risk ratio = 1.34), although it was not as clear in the divided-attention trial of the IB Cross task (Near: 84.2%, Far: 78.2%, *χ* ^*2*^ *(1)* = 1.09, *p* = .296, risk ratio = 1.08).

Study 2 provides one of the first opportunities to explore whether people who notice an unexpected object in one task are more likely to notice one in another task. If inattentional blindness is a stable personality trait across situations and paradigms, noticing on our two inattentional blindness tasks should be correlated. If, however, noticing is a stochastic process rather than a stable individual difference, noticing on one task may be unrelated to noticing on another. Data from two participants who inadvertently were assigned to different conditions in the two inattentional blindness tasks were excluded from this analysis, leaving a total of 172 participants. The association between noticing on one task and noticing on the other was small (*r*_*φ*_ = .13, 95% CI:-.02 to .27), and the confidence interval included 0. Apparently, the tendency to notice an unexpected object in one task does not strongly predict the ability to notice an unexpected object in another task. This finding is even more surprising given the fact that our participants were divided into two experimental conditions (Near, Far) that entailed significantly different inattentional blindness rates. Half of the participants, namely those assigned to the Near condition, had a higher chance of noticing the unexpected object in both inattentional blindness tasks. Accordingly, the weak relationship between the two different inattentional blindness tasks might still be overestimated. Calculating the relationship between noticing on one task and noticing on the other separately for the two conditions revealed even smaller or non-existent relationships (Near: *r*_*φ*_ = .08, 95% CI:-.13 to .28; Far: *r*_*φ*_ = .01, 95% CI:-.21 to .23). When interpreting these results, one has to keep in mind that the number cases included in the analyses were much smaller for the separated analyses. Note, that these results were obtained despite the fact that the preconditions for finding a positive relationship between the two inattentional blindness tasks were nearly optimal in this study: The noticing rates in the critical trial of the NEAR condition were nearly identical for IB Motion and IB Cross, as were the noticing rates in the FAR condition.

As in Study 1, we hypothesized that working memory capacity would predict noticing in the Near condition of each inattentional blindness task and that attention breadth would predict noticing in the Far condition of each task. Consistent with our predictions and with the results of Study 1, noticing in the Near condition of the IB Cross task was associated with the composite working memory measure, the 2-Back-Spatial task, and Aospan (see Table 4). Note that none of the p values were corrected for multiple tests because they were part of our pre-registered plan. Consequently, the few significant associations should be treated as tentative given that they are not particularly large. Noticing in the Near condition of the IB Motion task was not significantly correlated with the individual memory measures or the composite measure (see Table 5). Although working memory capacity is somewhat associated with noticing of central unexpected objects in a static task, it does not appear to drive noticing in a more dynamic task.

**Table 4:** Correlations (point biserial) among noticing of an unexpected object in IB Cross (all participants, Near condition, Far condition) and the cognitive tests; Study 2.

	ALL		NEAR		FAR	
	Notice	Notice	Notice	Notice	Notice	Notice
	(critical)	(divAtt)	(critical)	(divAtt)	(critical)	(divAtt)
Working Memory	**.18**	.05	**.26**	.03	.15	.08
	**[.04, .32]**	[-.10, .19]	**[.06, .44]**	[-.17, .23]	[-.06, .35]	[-.13, .29]
	N = 182	N = 182	N = 95	N = 95	N = 87	N = 87
2-Back-Identity	.04	.01	.01	-.01	.11	.03
	[-.11, .18]	[-.14, .16]	[-.19, .21]	[-.21, .19]	[-.10, .31]	[-.18, .24]
	N = 182	N = 182	N = 95	N = 95	N = 87	N = 87
2-Back-Spatial	**.15**	.05	**.26**	.00	.08	.10
	**[.00, .29]**	[-.10, .19]	**[.06, .44]**	[-.20, .20]	[-.13, .29]	[-.11, .30]
	N = 181	N = 181	N = 94	N = 94	N = 87	N = 87
Aospan	**.20**	.05	**.30**	.06	.14	.04
	**[.06, .34]**	[-.10, .19]	**[.11, .47]**	[-.14, .26]	[-.08, .34]	[-.17, .25]
	N = 182	N = 182	N = 95	N = 95	N = 87	N = 87
Attention Breadth	.12	.13	.09	.05	.12	.19
	[-.03, .26]	[-.02, .27]	[-.11, .29]	[-.15, .25]	[-.09, .32]	[-.02, .39]
	N = 182	N = 182	N = 95	N = 95	N = 87	N = 87
BoA	.10	.07	.03	-.06	.13	.18
	[-.05, .24]	[-.08, .21]	[-.17, .23]	[-.26, .14]	[-.08, .33]	[-.03, .38]
	N = 182	N = 182	N = 95	N = 95	N = 87	N = 87
UFOV	.10	**.15**	.11	.14	.08	.16
	[-.05, .24]	**[.01, .29]**	[-.09, .31]	[-.06, .33]	[-.13, .29]	[-.05, .36]
	N = 182	N = 182	N = 95	N = 95	N = 87	N = 87
Flanker	.13	.04	.17	.06	.08	.01
	[-.02, .27]	[-.11, .19]	[-.03, .36]	[-.14, .26]	[-.13, .29]	[-.20, .22]
	N = 181	N = 181	N = 94	N = 94	N = 87	N = 87
Navon	-.08	.10	-.09	.05	-.10	.13
	[-.22, .07]	[-.05, .24]	[-.29, .11]	[-.15, .25]	[-.30, .11]	[-.08, .33]
	N = 182	N = 182	N = 95	N = 95	N = 87	N = 87
Navon-Switchspeed	-.03	-.09	-.11	-.14	.04	-.04
	[-.18, .12]	[-.23, .06]	[-.31, .09]	[-.33, .06]	[-.17, .25]	[-.25, .17]
	N = 182	N = 182	N = 95	N = 95	N = 87	N = 87
CFQ	-.10	-.07	-.05	-.08	-.17	-.06
	[-.24, .05]	[-.21, .08]	[-.25, .15]	[-.28, .12]	[-.37, .04]	[-.27, .15]
	N = 182	N = 182	N = 95	N = 95	N = 87	N = 87

*Note*. In addition to the single cognitive measures the composite working memory measure and the composite attention breadth measure are depicted. The lower and upper bounds of the 95% confidence interval are shown in square brackets. *N* = number of cases contributing to each correlation. Correlations that are significant (α = 0.05; two-tailed) appear in bold, although the alpha level has not been corrected for multiple tests.

**Table 5:** Correlations (point biserial) among noticing of an unexpected object in IB Motion (all participants, Near condition, Far condition) and the cognitive tests; Study 2.

	ALL		NEAR		FAR	
	Notice	Notice	Notice	Notice	Notice	Notice
	(critical)	(divAtt)	(critical)	(divAtt)	(critical)	(divAtt)
Working Memory	-.10	-.06	-.03	-.16	-.16	.05
	[-.23, .04]	[-.20, .08]	[-.23, .17]	[-.35, .05]	[-.35, .05]	[-.16, .25]
	N = 189	N = 189	N = 96	N = 96	N = 93	N = 93
2-Back-Identity	-.07	-.07	-.04	-.10	-.09	-.04
	[-.21, .07]	[-.21, .07]	[-.24, .16]	[-.30, .10]	[-.29, .12]	[-.24, .17]
	N = 188	N = 188	N = 96	N = 96	N = 92	N = 92
2-Back-Spatial	-.10	-.04	-.08	-.19	-.07	.13
	[-.24, .04]	[-.18, .10]	[-.28, .12]	[-.38, .01]	[-.27, .14]	[-.08, .33]
	N = 188	N = 188	N = 95	N = 95	N = 93	N = 93
Aospan	-.06	-.02	.05	-.06	-.18	.02
	[-.20, .08]	[-.16, .12]	[-.15, .25]	[-.26, .14]	[-.37, .03]	[-.18, .22]
	N = 189	N = 189	N = 96	N = 96	N = 93	N = 93
Attention Breadth	-.11	-.03	-.02	.03	**-.25**	-.11
	[-.25, .03]	[-.17, .11]	[-.22, .18]	[-.17, .23]	**[-.43, -.05]**	[-.31, .10]
	N = 189	N = 189	N = 96	N = 96	N = 93	N = 93
BoA	-.04	-.03	-.03	-.02	-.12	-.08
	[-.18, .10]	[-.17, .11]	[-.23, .17]	[-.22, .18]	[-.32, .09]	[-.28, .13]
	N = 189	N = 189	N = 96	N = 96	N = 93	N = 93
UFOV	**-.15**	-.02	-.01	.06	**-.32**	-.11
	**[-.29, -.01]**	[-.16, .12]	[-.21, .19]	[-.14, .26]	**[-.48, -.11]**	[-.31, .10]
	N = 189	N = 189	N = 96	N = 96	N = 93	N = 93
Flanker	.07	.06	.04	.10	.10	.01
	[-.07, .21]	[-.08, .20]	[-.16, .24]	[-.10, .30]	[-.11, .30]	[-.19, .21]
	N = 188	N = 188	N = 95	N = 95	N = 93	N = 93
Navon	-.02	.05	.02	.02	-.05	.08
	[-.16, .12]	[-.09, .19]	[-.18, .22]	[-.18, .22]	[-.25, .16]	[-.13, .28]
	N = 189	N = 189	N = 96	N = 96	N = 93	N = 93
Navon-Switchspeed	.07	.11	-.03	.03	.15	.15
	[-.07, .21]	[-.03, .25]	[-.17, .11]	[-.27, .23]	[-.06, .34]	[-.06, .34]
	N = 189	N = 189	N = 96	N = 96	N = 93	N = 93
CFQ	.08	**.14**	-.04	.16	.20	.13
	[-.06, .22]	**[-.00, .28]**	[-.24, .16]	[-.04, .35]	[-.00, .39]	[-.08, .33]
	N = 189	N = 189	N = 96	N = 96	N = 93	N = 93

*Note*. In addition to the single cognitive measures the composite working memory measure and the composite attention breadth measure are depicted. The lower and upper bounds of the 95% confidence interval are shown in square brackets. *N* = number of cases contributing to each correlation. Correlations that are significant (α = 0.05; two-tailed) appear in bold, although the alpha level has not been corrected for multiple tests.

**Fig 4:**
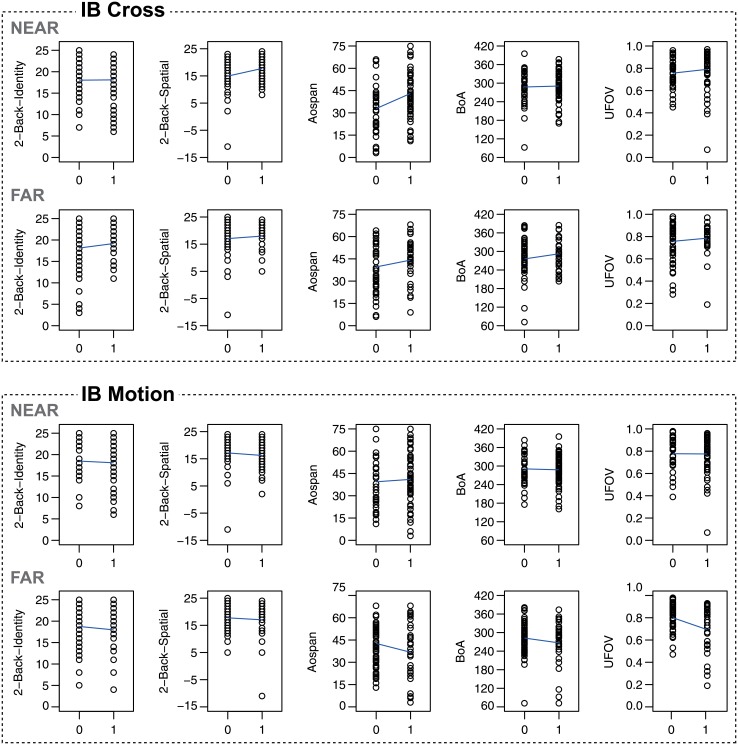
Scatterplots for Study 2. Scatter plots of the relationships between inattentional blindness (0 = miss, 1 = notice) and the working memory and attention breadth measures in Study 2. The plots were prepared separately for IB Cross and IB Motion and for the Near and the Far condition. The y-axes depict the test scores for each measure as described in the method section. Each circle represents a single participant. The blue lines depict the linear regression lines for each relationship.

Inconsistent with our prediction that people with greater attention breadth would be more likely to notice unexpected objects in a spatially-driven inattentional blindness task, noticing in the Far condition of the IB Cross task was largely unrelated to measures of attention breadth. And, contrary to our prediction, noticing in the Far condition of the IB Motion task was *negatively* associated with performance on the UFOV and the composite attention breadth measure. Combined with the lack of a correlation in Study 1, these findings provide little support for the idea that a wider attentional focus increases the likelihood of noticing a spatially distant unexpected object. Scatter plots of the relationships between inattentional blindness and the working memory and attention breadth measures are depicted in Fig 4 separately for IB Cross and IB Motion and separately for the Near and the Far condition.

As in Study 1, we conducted binary logistic regressions separately for the Near and Far conditions of each inattentional blindness task, predicting noticing from the composite working memory measure, the composite attention breadth measure, the Navon task, and the CFQ. The working memory and attention breadth measures were entered first, with the other measures entered in a second block. For the IB Motion task (see Table 6), none of the individual measures significantly predicted noticing in either condition. Predicting noticing using both working memory and attention breadth did not significantly improve the model. Including the Navon measure and CFQ in the second block did improve the fit for the Far condition (although the second block on its own did not significantly improve the model).

**Table 6:** Results of binary logistic regression with simultaneous entry in two blocks (SE in parentheses) Study 2, IB Motion.

NEAR						
	Variables	*B* (*SE*)	Wald	Exp(*B*)	Exp(*B*) lower	Exp(*B*) upper
	Constant*	0.65 (0.22)	8.99	1.91		
	Working Memory	-0.08 (0.30)	0.08	0.92	0.52	1.64
	Attention Breadth	-0.04 (0.29)	0.02	0.96	0.55	1.68
	*R* ^*2*^ = .*00 (Cox & Snell)*, *Model*: *χ* ^*2*^ *(2)* = *0*.*12*, *p* = .*94*					
	Constant	0.96 (0.91)	1.12	2.61		
	Working Memory	-.07 (0.31)	0.05	0.93	0.51	1.71
	Attention Breadth	-.04 (0.29)	0.02	0.96	0.55	1.68
	Navon	0.00 (0.02)	0.01	1.00	0.97	1.04
	CFQ	-0.01 (0.02)	0.15	0.99	0.96	1.03
	*R* ^*2*^ = .*00 (Cox & Snell)*, *Model*: *χ* ^*2*^ *(4)* = *0*.*27*, *p* = .*99*					
FAR						
	Variables	*B* (*SE*)	Wald	Exp(*B*)	Exp(*B*) lower	Exp(*B*) upper
	Constant*	-0.62 (0.23)	7.49	0.54		
	Working Memory	-0.17 (0.35)	0.23	0.85	0.43	1.68
	Attention Breadth	-0.52 (0.28)	3.49	0.60	0.35	1.03
	*R* ^*2*^ = .*06 (Cox & Snell)*, *Model*: *χ* ^*2*^ *(2)* = *5*.*99*, *p* = .*05*					
	Constant*	-1.99 (0.89)	5.05	0.14		
	Working Memory	-0.12 (0.36)	0.10	0.89	0.44	1.81
	Attention Breadth	-0.52 (0.28)	3.38	0.60	0.34	1.04
	Navon	-0.02 (0.02)	0.77	0.99	0.95	1.02
	CFQ	0.03 (0.02)	3.10	1.03	1.00	1.07
	*R* ^*2*^ = .*10 (Cox & Snell)*, *Model*: *χ* ^*2*^ *(4)* = *9*.*59*, *p <* .*05*					

*Note*. Both regressional analyses (Near, Far) were performed in two separate blocks. The variables of the first block are depicted first and the variables of the whole model, including the second block, are depicted below it. The upper and the lower bounds of the 95% confidence interval of Exp(*B*) are depicted as well.

*p < .05

For the IB Cross task (see Table 7), adding the working memory and attention breadth predictors significantly improved the model in the Near condition, explaining about 7% of the variance. The working memory measure was the only significant predictor of noticing, and including Navon and CFQ in a second block did not improve the model significantly. In fact, using all four predictors did not provide a significant fit. For the Far condition, none of the predictors individually or jointly predicted noticing.

**Table 7:** Results of binary logistic regression with simultaneous entry in two blocks (SE in parentheses) Study 2, IB Cross.

NEAR						
	Variables	*B* (*SE*)	Wald	Exp(*B*)	Exp(*B*) lower	Exp(*B*) upper
	Constant*	0.56 (0.22)	6.34	1.76		
	Working Memory*	0.72 (0.31)	5.40	2.05	1.12	3.75
	Attention Breadth	0.10 (0.29)	0.13	1.11	0.63	1.95
	*R* ^*2*^ = .*07 (Cox & Snell)*, *Model*: *χ* ^*2*^ *(2)* = *6*.*50*, *p <* .*05*					
	Constant	1.20 (0.96)	1.59	3.33		
	Working Memory*	0.72 (0.32)	4.98	2.05	1.09	3.85
	Attention Breadth	0.11 (0.29)	0.15	1.12	0.63	1.97
	Navon	-0.00 (0.02)	0.03	1.00	0.96	1.03
	CFQ	-0.01 (0.02)	0.46	0.99	0.95	1.03
	*R* ^*2*^ = .*07 (Cox & Snell)*, *Model*: *χ* ^*2*^ *(4)* = *6*.*99*, *p* = .*14*					
FAR					
	Variables	*B* (*SE*)	Wald	Exp(*B*)	Exp(*B*) lower	Exp(*B*) upper
	Constant*	-0.68 (0.23)	8.38	0.51		
	Working Memory	0.36 (0.37)	0.94	1.43	0.70	2.94
	Attention Breadth	0.19 (0.30)	0.39	1.21	0.67	2.18
	*R* ^*2*^ = .*03 (Cox & Snell)*, *Model*: *χ* ^*2*^ *(2)* = *2*.*41*, *p* = .*30*					
	Constant	0.62 (0.96)	0.41	1.85		
	Working Memory	0.35 (0.38)	0.87	1.42	0.68	2.98
	Attention breadth	0.13 (0.31)	0.17	1.14	0.62	2.08
	Navon	-0.01 (0.02)	0.50	0.99	0.95	1.02
	CFQ	-0.03 (0.02)	1.52	0.97	0.93	1.02
	*R* ^*2*^ = .*05 (Cox & Snell)*, *Model*: *χ* ^*2*^ *(4)* = *4*.*77*, *p* = .*31*					

*Note*. Both regressional analyses (Near, Far) were performed in two separate blocks. The variables of the first block are depicted first and the variables of the whole model, including the second block, are depicted below it. The upper and the lower bounds of the 95% confidence interval of Exp(*B*) are depicted as well.

*p < .05

Given that we did not make separate predictions for the influence of the Navon-Switchspeed task or the Flanker task for the Near and Far conditions, we combined across these conditions in each task and performed two separate regressions predicting noticing in each inattentional blindness task from these measures (Table 8). Neither measure individually or jointly predicted noticing in the IB Motion task or the IB Cross task. Thus, again, individual differences in the ability to actively inhibit known distractors in a Flanker task were not related to the tendency to miss an unexpected object. This finding lends further support to the idea that fundamentally different cognitive processes underlie the inhibition of known distraction and the unawareness of *unexpected* stimulation [29,30].

**Table 8:** Results of the binary logistic regression with simultaneous entry (SE in parantheses); Study 2.

IB Cross						
	Variables	*B* (*SE*)	Wald	Exp(*B*)	Exp(*B*) lower	Exp(*B*) upper
	Constant	-0.53 (0.45)	1.43	0.59		
	Flanker	0.05 (0.03)	3.14	1.06	0.99	1.12
	Navon-Switchspeed	-0.00 (0.01)	0.04	1.00	0.97	1.02
	*R* ^*2*^ = .*02 (Cox & Snell)*, *Model*: *χ* ^*2*^ *(2)* = *3*.*32*, *p* = .*19*					
IB Motion						
	Variables	*B* (*SE*)	Wald	Exp(*B*)	Exp(*B*) lower	Exp(*B*) upper
	Constant	-0.55 (0.44)	1.56	0.58		
	Flanker	0.32 (0.30)	1.13	1.03	0.97	1.09
	Navon-Switchspeed	0.12 (0.01)	1.03	1.01	0.99	1.04
	*R* ^*2*^ = .*01 (Cox & Snell)*, *Model*: *χ* ^*2*^ *(2)* = *2*.*08*, *p* = .*35*					

*Note*. The upper and the lower bounds of the 95% confidence interval of Exp(*B*) are depicted as well. No variable had a *p* < .05.

Given the significant and relatively large overall effect of condition (Near, Far) we additionally calculated a regression model that included the Flanker task and the Navon-Switchspeed task as part of the main regression model separately for Near and Far. These additional analyses can be found at https://osf.io/mvwih/.

As noted in Study 1, the measures of attention breadth might not reflect the role of spatial attention in performing either the line-judgment task or the dynamic tracking task. Rather, both the BoA and UFOV measure the *maximum* ability to spread attention peripherally, not the default tendency to spread attention when performing a focused attention task. To assess the default tendency to focus attention broadly or narrowly, we included a Navon task, but individual differences in attention style did not predict noticing either. Taken together, these findings suggest a negligible role for individual differences in spatial attention in predicting noticing of unexpected objects. Moreover, the pattern of results suggests that individual differences in working memory capacity play a limited role (affecting performance only in the IB Cross task and not strongly) in predicting noticing, even when inattentional blindness is induced by solely placing demands on central cognitive resources. The pattern of results in Study 2 largely confirmed those of Study 1.

As in Study 1, individual differences in working memory performance were not associated with noticing the additional object in the divided-attention trial for either condition of either inattentional blindness task. To the extent that working memory capacity is associated with the ability to divide attention across multiple task demands, that ability does not enhance the ability to perform the primary task while also trying to detect a critical object. Although the strength of this relationship is limited by the relatively high noticing rates in the divided-attention trials, the data show no clear trend favoring such a link.

## General Discussion

The primary goal of these studies was to explore whether individual differences in attention and working memory predict inattentional blindness. More specifically, we tested whether inattentional blindness induced by attention to the wrong location would be predicted by differences in attention breadth and whether inattentional blindness induced by general resource limitations would be predicted by working memory differences (see [25,56] for the distinction between different types of inattentional blindness).

None of our cognitive measures reliably predicted spatially-induced inattentional blindness, regardless of whether the inattentional blindness task was static (Study 1 & 2) or dynamic (Study 2). Apparently, neither the maximum breadth of attention nor global/local attention biases predict spatial failures of awareness. In both studies, working memory measures did predict noticing of a spatially proximal unexpected object in a static inattentional blindness task, albeit weakly. However, working memory differences did not predict noticing in a dynamic task even though tracking multiple objects simultaneously should place a heavier demand on central cognitive resources than a static line-judgment task [57,58]. Additionally, working memory did not predict noticing on the divided-attention trials of that same task even though we might expect working memory capacity to contribute to the ability to look for the critical object while simultaneously performing the primary task.

Taken together, the link between individual differences in working memory and noticing of unexpected objects is fairly small and does not generalize to inattentional blindness tasks and situations for which working memory might be expected to play a role. This pattern raises the possibility that such differences are less important for more ongoing, dynamic attention-demanding situations like those we might experience in the real world.

Most prior studies of individual differences in noticing have tried to predict noticing of a single unexpected object based on personality or cognitive ability measures. Study 2 was among the first to compare noticing by the same individuals in two inattentional blindness tasks. To our knowledge, only two other studies examined inattentional blindness within the same individuals across multiple instances. One used a small sample (N = 36) to test whether familiarity with and prior experience in an inattentional blindness study would affect noticing [50] and the other investigated inattentional blindness within the same task by inducing incorrect expectations about the nature of the unexpected object [51]. Neither analyzed the stability of inattentional blindness within an individual across events.

The challenge in measuring inattentional blindness twice is that, for the second task, participants know that an additional object might appear. Thus, the additional object in the second task is not entirely unexpected, meaning that it might not measure inattentional blindness. For several reasons, we believe that our second task did measure inattentional blindness and that our participants did not anticipate the unexpected object in the second task: (1) The two tasks were embedded in a larger battery of cognitive tasks; (2) The tasks looked different and the primary task in each placed different demands on participants; (3) Few participants reported having expected anything unexpected to appear on the second task; and (4) Noticing rates in the static task were comparable across studies, suggesting that expectations did not alter noticing rates substantially.

Apparently, the tendency to notice unexpected objects is not a stable and robust individual difference that applies in a general way across a variety of tasks; noticing of an unexpected object in one task only weakly predicted noticing in the other. Nonetheless, even if proneness to inattentional blindness is not a stable trait that applies across a range of situations and tasks, individual differences may reliably predict noticing for a given task. For example, distinct individual differences factors might affect noticing in static and dynamic tasks.

Given that it is not possible to measure inattentional blindness repeatedly using the same task (but see [51]) because participants will expect that object to appear again in subsequent trials, the search for individual differences that predict susceptibility to inattentional blindness in each task separately might prove fruitful. However, we found little evidence that individual differences predict noticing in either task; only the Near condition of the static cross task showed a consistent relationship with some measures of working memory, and those memory measures did not predict noticing in other conditions (e.g., divided attention, dynamic tasks) when we might expected them to. Given the lack of evidence that individual differences in cognitive abilities consistently predict noticing, it appears that noticing is affected more by the task demands and the situation (e.g., [8,32,59–61]; but see [16] for evidence that expert knowledge in a domain might influence noticing rates within that domain) than by stable individual differences.

### Limitations of the Current Study

Our goal was to measure individual differences in the ability to notice unexpected objects, not the ability to perform the primary task. Equating primary-task performance is perhaps the best way to isolate individual differences in noticing from individual differences in primary-task performance. Although other studies suggest that individual difference in primary-task performance do not predict noticing [32], equating performance across participants might have eliminated some systematic variation in noticing that otherwise would be predicted by cognitive measures like working memory or attention breadth.

Although we found little relationship between noticing and other cognitive tasks, our ability to detect such correlations might have been limited by the reliability of those measures. Several of our measures (2-Back-Identity, Flanker, and Navon-Switchspeed) had low test-retest reliabilities (based on our small sample of retested participants), meaning that their usefulness as predictive measures might be relatively low. Still, even those measures that showed high reliability, including the main measures of attention breadth and working memory, were largely unrelated to noticing.

Assessing the reliability of individual inattentional blindness tasks is problematic due to the single-trial nature of the phenomenon [29]; once participants are asked about an unexpected object, that critical object will not be entirely unexpected on future trials. Yet, inattentional blindness has been used to study individual differences (e.g., [19,23,29], and noticing rates do vary systematically as a function of transient factors such as arousal [62], mood [63], and cognitive load [11]. Moreover, noticing varies predictably with task demands (e.g., [5,9,64]) and differs across groups of people with specific skills or abilities [15–17]. Thus, even if we cannot directly confirm the reliability of a single inattentional blindness task, performance on that task is predictable from other factors.

Another approach to exploring the reliability of an inattention blindness task might be to vary expectations over the course of a study; when the properties of a critical object vary over the course of a study, wrong expectations can produce repeated inattention blindness in the same task [51]. Although that new approach does not yield a measure of reliability for a particular unexpected object, it could provide a means to assess the reliability of noticing in a given inattentional blindness task. However, the tasks demands differ from the standard inattention blindness situation because failures to notice are driven by having the wrong expectations rather than by having no expectation at all.

Our ability to predict noticing from individual-differences measures might have been hampered by a restricted range of performance on the cognitive tasks. Even though all of our measures did show substantial between-subject variability, meaning we had sufficient variability to detect associations among them, our sample consisted of university students, and ideally, these findings should be replicated with a non-student population. Performance on many of the cognitive tasks we used [65–67] as well as susceptibility to inattentional blindness [17] changes across the lifespan, and the associations among them might change as well. For example, a preliminary study with a small sample of older adults observed a link between fluid intelligence and inattentional blindness [68]. It remains unclear, however, whether this finding depends on the specific age range of the sample or on the cognitive measure used. Given the instability of correlations in small samples, this individual-difference finding should be verified with a larger sample.

## Conclusion

Individual differences in cognitive abilities such as attention breadth and working memory do not reliably predict noticing of unexpected objects. Moreover, working memory and attention breadth did not separately predict noticing in centrally- and spatially-induced inattentional blindness. Although working memory capacity was weakly associated with noticing in the central condition of a static inattentional blindness task, it did not predict noticing in the comparable condition of a dynamic task, suggesting that working memory does not predict noticing of unexpected objects in general. And, to the extent that it does predict noticing, the association is relatively weak. The minimal correlation between noticing in our two inattentional blindness tasks suggests that the ability to notice unexpected objects in general is not a stable individual-difference trait. Consequently, inattentional blindness appears to be driven more by situational and task factors, or even by chance, than by individual-differences variables.
